# Multiple Sclerosis Treatment in the COVID-19 Era: A Risk-Benefit Approach

**DOI:** 10.3390/neurolint14020030

**Published:** 2022-04-15

**Authors:** Paolo Immovilli, Nicola Morelli, Chiara Terracciano, Eugenia Rota, Elena Marchesi, Stefano Vollaro, Paola De Mitri, Domenica Zaino, Veronica Bazzurri, Donata Guidetti

**Affiliations:** 1 Neurology Unit, Emergency Department, Guglielmo da Saliceto Hospital, 29121 Piacenza, Italy; nicola.morelli.md@gmail.com (N.M.); c.terracciano@ausl.pc.it (C.T.); e.marchesi@ausl.pc.it (E.M.); s.vollaro@ausl.pc.it (S.V.); p.demitri@ausl.pc.it (P.D.M.); zainomimma@gmail.com (D.Z.); v.bazzurri@ausl.pc.it (V.B.); d.guidetti@ausl.pc.it (D.G.); 2 Radiology Unit, Radiology Department, Guglielmo da Saliceto Hospital, 29121 Piacenza, Italy; 3 Neurology Unit, San Giacomo Hospital, 15067 Novi Ligure, Italy; eugenia.rota.md@gmail.com

**Keywords:** multiple sclerosis, COVID-19, SARS-CoV-2, anti-CD20, fingolimod, ocrelizumab, S1P-modulators, DMD, MS therapy-related risks, vaccination

## Abstract

The COVID-19 pandemic poses an ongoing global challenge, and several risk factors make people with multiple sclerosis (pwMS) particularly susceptible to running a severe disease course. Although the literature does report numerous articles on the risk factors for severe COVID-19 and vaccination response in pwMS, there is a scarcity of reviews integrating both these aspects into strategies aimed at minimizing risks. The aim of this review is to describe the risk of vulnerable pwMS exposed to severe acute respiratory syndrome coronavirus 2 (SARS-CoV-2), the issues related to the SARS-CoV-2 vaccine and to evidence possible future strategies in the clinical management of pwMS. The authors searched for papers on severe COVID-19 risk factors, SARS-CoV-2 vaccination and people with multiple sclerosis in support of this narrative literature review. We propose a multilevel strategy aimed at: the evaluation of risk factors for severe COVID-19 in people with multiple sclerosis, identifying the most appropriate vaccination schedule that is safe for people on disease-modifying drugs (DMDs) and a strict follow-up of high-risk people with multiple sclerosis to allow for the prompt administration of monoclonal antibodies to manage COVID-19 risks in this patient population.

## 1. Introduction

Multiple sclerosis (MS) is a demyelinating disease of the central nervous system, characterized by inflammation and early axonal damage [1], and it is the leading cause of disability in young adults [2]. MS is a multifactorial disease caused by a complex interplay between genetic and ambiental risk factors. Some genes encoding for immune system components lead to a higher risk of developing MS and other acquired risk factors (vitamin D plasma level, solar light exposition, salt intake, the smoking habit and some infections, etc.) may also increase the risk [3,4,5]. Moreover, it seems that the Epstein–Barr virus infection is a “necessary but not sufficient” factor for the development of MS [6]. 

MS is characterized by a wide range of motor, behavioral and cognitive symptoms [7], reflecting the “functional systems” involved by inflammation, demyelination and neurodegeneration. Its diagnosis, according to the 2017 revised MacDonald criteria [8], is based on the demonstration of “a dissemination in space and time” of typical multiple sclerosis lesions evidenced by brain and spinal cord magnetic resonance imaging [9,10].

The disease-modifying drugs (DMDs) used to treat MS comprise a range of molecules and monoclonal antibodies, which have a wide spectrum of efficacy and adverse events [11,12,13]. Although most MS DMDs affect the immune system, some of them also affect the neurodegenerative metabolic pathways, e.g., the nuclear factor erythroid-2-related factor 2 (NRF2)-Kelch-like ECH-associated protein 1 (KEAP1) signaling, an enzyme involved in the pathogenesis of MS [14].

The coronavirus disease (COVID-19) pandemic has put enormous strain on healthcare organizations, medical resources and emergency medical services [15]. Primary care bears a particularly high burden, and the management of multiple sclerosis (MS) involves reconfiguring patient care so as to take into account specific aspects. Indeed, additional risk factors for severe COVID-19 must be considered for people with multiple sclerosis (pwMS), i.e., progressive disease, disability and immunomodulating/suppressive treatment [16]. Moreover, vaccination is not without certain implications for pwMS, in as much as MS treatment may not only interact with the vaccine response, but the vaccine may also have an effect on the MS course. There is a complex interplay between COVID-19 pathogenesis and the psychological consequences of the pandemic. The hallmark of COVID-19 pathogenesis is the cytokine storm, which may lead to severe pneumonia, stroke [17], pulmonary thromboembolism and critical illness. Indeed, COVID-19-associated psychological stress is one of the major consequences of the pandemic and could interact with inflammatory and degenerative processes in the central nervous system [18]. Moreover, COVID-19 fear has been associated with amygdala activation [19], and the resilience to psychological consequences of the pandemic seems to be a crucial factor to prevent negative sequelae [20].

Even neurologists had to face with the fears associated with COVID-19, which led to concerns that modified neurologists’ attitude toward prescribing DMDs [21]. They must now take into account the pros and cons of a severe COVID-19 and the risk of the disability related to MS under-treatment, the risk of a severe COVID-19 and propose the most appropriate vaccination strategy to be used, depending on the treatment regime. 

Numerous epidemiological studies have described the risk factors involved in severe COVID-19 in MS patients [22,23,24,25]. Other studies have investigated the humoral and cellular immune response to vaccines [26,27]. Apart from these studies, although there are some systematic reviews on COVID-19 in MS patients and recent studies on breakthrough infection in vaccinated MS patients [28], the publications that pool all these aspects aimed at identifying future strategies in the management of pwMS in the COVID-19 era are limited. 

Therefore, this narrative review aims at describing the risk vulnerable pwMS run when exposed to severe acute respiratory syndrome coronavirus 2 (SARS-CoV-2), the issues related to SARS-CoV-2 vaccine and proposing possible future strategies for the clinical management of pwMS.

## 2. Materials and Methods

The decision to write this narrative review was prompted by the aim of describing the different aspects of SARS-CoV-2 infection in pwMS. 

The authors searched for the terms “COVID-19 and Multiple Sclerosis” in PubMed from 2020 to 2022 and identified 756 papers. This is a narrative review and, for the purpose of the study, the authors selected the most relevant papers reporting on the risk pwMS run of developing a severe COVID-19 and the management of their SARS-CoV-2 vaccination. Preprints from MEDRxiv and guidelines published by regulatory agencies, scientific societies and the Italian Ministry of Health have also been included in this review. 

The papers selected included studies on different aspects of COVID-19 in pwMS targeted at reporting a synthesis of current knowledge and describing possible future strategies to reduce the risks related to SARS-CoV-2 infection in pwMS.

The following fields of research were taken into consideration:

- COVID-19 mortality and risk factors for severe COVID-19 in pwMS;
- vaccine response (humoral and cellular) in pwMS;
- breakthrough infection in pwMS;
- the effect of SARS-CoV-2 vaccination on MS.

Although this review included studies where the diagnosis of COVID-19 was based on the patients’ clinical characteristics and a positive swab test for SARS-CoV-2, data on swabs were not mandatory, as during the first wave of the pandemic, the incidence overwhelmed the testing capacity in many countries. The studies selected on vaccination included only those that reported on BNT162b2 and mRNA-1273 vaccines.

A collective author decision, based on a consensus upon papers’ relevance and quality, was made on the final choice of the 23 most relevant papers, as a systematic review and a meta-analysis were beyond the scope of this narrative review. 

A synthesis of the most relevant findings was then created and discussed.

## 3. Results

### 3.1. COVID-19 Mortality

COVID 19 mortality during the pandemic is estimated by the case fatality rate. This rate is highly variable [29] and depends on many factors, some of which can be related to the patient (risk factors for severe COVID-19), to the SARS-CoV-2 virus, and others to the pandemic phase. The factors linked to the patients include age and some diseases (cardiovascular, cerebrovascular, lung, chronic kidney disease, obesity, cancer, immunosuppression, etc.). All these factors are under the “umbrella term” severe COVID-19 risk factors. The core issue is whether MS and DMDs are risk factors for pwMS [22,23,24,25].

Variants and mutations are linked to the virus and may determine different infectivity and mortality, depending on the variant. The factors linked to the pandemic phase are the COVID-19 incidence rate and the intensive care unit (ICU) hospitalization rate. 

There is an association between mortality and incidence [15] and an inverse association between mortality and the rate between hospitalization in an ICU and/or medical wards [30]. The lockdown reduced social contacts and viral spreading, and the increase in the availability of ICU beds allowed intensive care to be provided to a larger population. Unfortunately, the long-term social costs of these interventions are very high and not sustainable. 

Although the COVID-19 crude mortality rate (CDR) for pwMS and the general population did not differ greatly, pwMS are mostly female and young. Therefore, when mortality was corrected for gender and age, it was higher in pwMS, particularly when they had significant disability and a progressive disease course [31]. Furthermore, Prosperini et al. carried out a pooled analysis of cohort studies and reported a 24% increased risk of death from COVID-19 in patients with MS. Additional risk factors for higher mortality were age, comorbidity, a progressive disease course, anti-CD20 therapy; interferon and teriflunomide administration were inversely associated with mortality [32]. 

### 3.2. Severe COVID-19 Risk Factors in pwMS

The most common COVID-19 symptoms in pwMS were asthenia, cough, fever, headache, anosmia, ageusia, dyspnea, digestive disorders and/or dizziness. Cough and dyspnea were more frequently observed in severe cases and headache and anosmia in outpatient cases [23]. SARS-CoV-2 infection has a wide range of severity, from asymptomatic infection to fatal acute respiratory distress syndrome (ARDS). Epidemiological studies frequently split the disease severity into a three-level variable: (1) mild-severity disease, characterized by no need for hospitalization and the absence of pneumonia; (2) intermediate-severity disease, characterized by the need for hospitalization and/or the presence of pneumonia; (3) severe COVID-19, characterized by death or ICU admission [22]. The Covisep study group described SARS-CoV-2 infection severity with a seven-level variable (the so-called “COVID-19 severity score”) [23], but in a further pooled analysis of French and Italian data, COVID-19 severity was described as a three-level variable [16]. 

Risk factors for severe COVID-19 were older age, male gender, an Extended Disability Status Scale (EDSS) score of >3, comorbidities (i.e., cardiac, obesity, etc.), having been administered methylprednisolone in the month before infection, anti-CD20 [16,22,23,24,25] and a progressive MS course. Interferon was associated with a better outcome [32]. Table 1 reports the main risk factors for severe COVID-19 in pwMS.

### 3.3. Vaccine Response in pwMS

DMDs can affect vaccine efficacy in pwMS, as reported by some papers. The first one was published by Achiron et al., who observed a reduced vaccine humoral response in patients on ocrelizumab and fingolimod [33]. Then, the Italian CovaxiMS group reported a reduced humoral response in patients on fingolimod and anti-CD20, and a better vaccination response was associated with higher pre-vaccination antibody levels and the use of mRNA-1273 (Moderna) vaccine. A better humoral response to vaccination was observed in pwMS treated with ocrelizumab when it was inoculated later, after the last ocrelizumab infusion, and higher antibody titers were observed when the lymphocyte count was higher in patients on fingolimod [34]. 

Other studies investigated the T-cell immune response and the humoral response together after vaccination, observing a reduction in both responses during fingolimod treatment and a reduction in humoral response along with an enhancement in T-cell response during anti-CD20 therapy [26,27].

The immune response to SARS-CoV-2 infection in pwMS treated with DMDs was similar to the vaccine response, i.e., the cellular response was still present at 13 months after COVID-19 in 59.5%, and the humoral response persisted up to 6 months in 81.1%. Patients on treatment with anti-CD20 had a lower antibody response, but severe COVID-19 and a longer time lapse since the last infusion was associated with a better humoral response. There was a cellular response in all the treatment groups [35]. Immune T-cell response to vaccination seems to be more important than antibodies in terms of prevention of death, hospitalization and severe COVID-19 [36].

Some authors reported a better vaccine response in pwMS on anti-CD20 treatment who did not have a complete B-cell depletion at vaccination, and there was a better response in pwMS treated with S1P modulators in the presence of a high lymphocyte count [37].

Table 2 summarizes the effect of each MS DMD on vaccine response and the risk of developing severe COVID-19.

### 3.4. The Effects of Vaccines on MS 

Di Filippo and Achiron investigated the relapse risk after Pfizer/BioNTech BNT162b2 vaccine in an Italian and Israeli cohort, reporting no increased risk of relapse activity [38,39]. However, there are some single-case reports and a case series of pwMS who had relapses after SARS-CoV-2 vaccination. Noteworthy is the case series reported by Nistri et al., where there was a temporal association between vaccination and MS relapses, although the study design made it impossible to establish whether this association was incidental or causative [40]. In this complex interplay between immune system stimulation and relapse risk, it must not be forgotten that single cases of MS relapses after COVID-19 have also been reported [41].

### 3.5. Breakthrough Infection

Research investigating the risk of COVID 19 in vaccinated pwMS treated with DMDs is ongoing, and the Italian CovaXiMS group published a pre-print reporting an association between low specific SARS-CoV-2 antibody titers and the risk of SARS-CoV-2 breakthrough infection [28]. The French COVISEP group described 18 cases of breakthrough infections; 13/18 were treated with antiCD-20, but only 1/13 was hospitalized for non-invasive mechanical ventilation. These findings are consistent with an increased risk of breakthrough infection but not for severe COVID-19 in pwMS vaccinated during anti-CD20 treatment [42].

### 3.6. Guidelines

There is a rapid evolution in the wealth of knowledge available on COVID-19, leading to continuous updates in the guidelines. Herein, we report on some of the most relevant statements made by the European Medical Agency [43], the Italian Ministry of Health [44] and the Italian Neurological Society [45]:

- SARS-CoV-2 vaccination is recommended in all pwMS, as they are very vulnerable patients and should be vaccinated as a priority;
- pwMS treated with injectables, dimethyl-fumarate, teriflunomide, azathioprine, S1P-modulators, natalizumab, cladribine should be vaccinated, whatever their therapy, but should be advised about the possibility of a reduced vaccine efficacy in the presence of lymphopenia;
- pwMS treated with mitoxantrone, cyclophosphamide, ocrelizumab, ofatumumab, rituximab, alemtuzumab should be given DMDs at least 4–6 weeks after the second vaccination and be vaccinated at least three months after the last infusion. With regard to the pandemic phase and disease activity, these time intervals are not mandatory.;
- the humoral response to the vaccination could be weak in pwMS treated with anti-CD20 or S1P-modulators;
- an interval of one month is recommended between an MS relapse and vaccination, as well as between steroid treatment and vaccination;
- vaccination is recommended for caregivers and pwMS relatives;
- an additional vaccine dose at least 28 days after the second dose is recommended in patients with immunodeficiency due to pharmacological treatment (ocrelizumab, rituximab, ofatumumab, S1P-modulators, alemtuzumab, cyclophosphamide, mitoxantrone, hematopoietic stem cell transplantation; cladribine, azathioprine, teriflunomide and dimethyl-fumarate when they induce a lymphopenia with a lymphocyte count of <800 cells/mm^3^ at vaccination);
- a booster vaccine dose is recommended at least 6 months after the second vaccination in pwMS treated with injectables, natalizumab, teriflunomide and dimethyl-fumarate, in the absence of lymphopenia;
- a fourth booster dose is recommended for those pwMS who were given a third additional vaccination because of drug-induced immunodeficiency.

## 4. Discussion

A risk and benefit evaluation of pwMS in the COVID-19 era has increased the complexity of the balance between the risk of disability accrual in undertreated MS and therapy-related risks. After the first high-efficacy treatment for MS (natalizumab) was approved in 2004, there was a voluntary withdrawal from the market due to two cases of progressive multifocal leukoencephalopathy only four months later. The drug was reintroduced onto the market with a black-box warning and a multilevel intervention to manage the risk–benefit profile at an individual level. 

Firstly, a risk stratification strategy was implemented (the so-called “stratify JCV test”) to clarify the risks at an individual level [46]. A surveillance MRI follow-up strategy was then proposed for an early diagnosis and, last but not least, an extended dose infusion schedule was adopted aimed at minimizing risks. 

The favorable risk–benefit profile of natalizumab is an example of a successful therapy risk management plan.

The fears involved with the COVID-19 outbreak could affect the neural pathways involved in the modulation of behavior. Indeed, it directly modulates amygdala activation [19], and social distancing indirectly modulates peri-personal space interaction neural pathways, determining autonomic reactions [47] and negative psychosocial effects [48]. 

At the beginning of the pandemic, the neurological community had strong reservations about administering DMDs to pwMS due the risk of severe COVID-19, and many neurologists were reluctant to begin therapy or switch it due to the fear of COVID-19 infection [21]. However, delaying treatment or changing one type of drug for another one with a higher efficacy may lead to MS undertreatment and, consequently, disability accrual.

A suitable strategy would be a risk management plan with multilevel interventions aimed at obtaining a better understanding of the risks of severe COVID-19 at an individual patient and drug level so as to minimize these risks through an appropriate immunization strategy, as well as monitoring for COVID-19 symptoms to allow for early treatment with antiviral drugs or monoclonal antibodies. 

There is an increased COVID-19 mortality rate in pwMS [31,32,49]. This makes MS “per se” an independent risk factor for the development of severe COVID-19. Additional risk factors include older age, comorbidities, progressive MS, high EDSS score, male gender, S1P modulators and anti-CD20 therapies. An evaluation of these risk factors can identify high-risk pwMS and protect them, i.e., through a complete immunization schedule or the administration of anti-SARS-CoV-2 monoclonal antibodies in the early phase of COVID-19. 

Any pwMS on anti-CD20 or S1P modulators are eligible for an adjunctive vaccination dose (i.e., a full third dose of mRNA 1273) [44], and studies investigating the effect of a fourth booster dose are ongoing. A strict follow-up should be scheduled for high-risk pwMS so as to decide whether or not to start early antiviral therapy and/or monoclonal antibody administration. DMDs other than anti-CD20 and S1P modulators had either no effect or a protective effect against the risk of severe COVID-19 in pwMS.

Data have promoted SARS-CoV-2 vaccination for pwMS, especially with Pfizer-BioNTech (BNT162b2) and Moderna mRNA (mRNA-1273), and no association was observed between vaccination and MS relapses in most studies [38,39], even if there are some case reports or case series on relapses after vaccination with Pfizer-BioNTech [40], as well as after COVID-19 infection [41]. Therefore, mRNA vaccines had a positive risk–benefit profile in pwMS.

Literature data reported no relevant effect on vaccine response for DMDs other than anti-CD 20 and fingolimod [33,34,37]. Although anti-CD20 tend to increase the risk of severe COVID-19, and both anti-CD20 and S1P-modulators weaken the vaccine response, these drugs are precious arms against MS, and undertreatment leads to the risk of disability accrual. We are of the opinion that it is more appropriate to use all DMDs without restriction and vaccinate with the best schedule for anti-CD20 and S1P modulators. 

So as to allow for an increase in the lymphocyte count, vaccination in a set time frame of two months with a reduced dose of S1P modulators should be considered for pwMS treated by S1P modulators with a low lymphocyte count. The appropriate dose of S1P modulators should then be administered 2 weeks after the second dose. Although this strategy would mean pwMS would have a couple of months of low dose treatment, it should enhance the vaccine response. We propose that pwMS on anti-CD20 be vaccinated between 4 to 6 months from the last anti-CD20 infusion, preferentially when B-cell depletion is recovering. Moreover, these pwMS should minimize their risk of infection by SARS-CoV-2 through the adoption of scrupulous hygiene practices, e.g., the use of PPE, such as facial KN95 masks and regular hand sanitizing.

Progressive pwMS, with comorbidities, older age and/or high EDSS score run a higher risk of severe COVID-19. Therefore, a careful risk/benefit assessment should be performed in this particular group so as to reduce relapses when still present or slow disability worsening, weighing these issues against the risks of specific therapy. 

We are aware that our review does have some limitations, the main one being the study design. That is, this is a narrative review without a systematic study collection or a meta-analysis, which limits the strength of the evidence [50]. However, it was decided to review various topics, i.e., severe COVID-19 risk and vaccination in pwMS, with the aim of proposing a comprehensive clinical strategy to manage pwMS in the COVID-19 era.

## 5. Conclusions

Briefly, we are of the opinion that, in light of the findings reported in the literature, although it is necessary to adapt the management of pwMS, this does not mean it should be compromised if the challenges still posed by COVID-19 are to be met. We suggest that a careful individual assessment of the risk of a severe COVID-19 infection be made for pwMS so as to manage this risk with the most appropriate vaccination schedule, chosen taking into consideration the DMD and that the risks involved in avoiding the administration of that DMD should be weighed against its benefits. 

## Figures and Tables

**Table 1:** Risk factors for severe COVID-19 pwMS.

Severe COVID-19 Risk Factors
Older age Male gender EDSS score of >3 Cardiac comorbidities Obesity Progressive MS course Administration of high doses of methylprednisolone in the month before infection
Anti-CD20 therapy

References: [2,4,5,6,13,25].

**Table 2:** DMDs effect on severe COVID-19 risk and vaccine response.

DMD	Severe COVID-19 Risk OR (95% IC)	Effects on Vaccine Response
Interferons	0.42 (0.18–0.99)	n.s. ^§^
Glatiramer acetate	n.s. ^§^	n.s. ^§^
Teriflunomide	Significant ^#^	n.s. ^§^
Dimethyl fumarate	n.s. ^§^	n.s. ^§^
S1P modulators	n.s. ^§^	Reduced humoral and cellular response
Cladribine	n.s. ^§^	n.s. ^§^
Anti-CD20	2.05 [1.39–3.02]	Reduced humoral response
Natalizumab	n.s. ^§^	n.s. ^§^
Alemtuzumab	n.s. ^§^	n.s. ^§^

^§^ n.s., not significant. ^#^ negative correlation between lethality and the percentage of patients under treatment with teriflunomide (*p*-value 0.035). References: [2,4,5,6,7,8,13,14,15,16,25].

## Data Availability

Not applicable.
